# Supplementary material for: Analysis of Porcine Pro- and Anti-Inflammatory Cytokine Induction by S. suis In Vivo and In Vitro
Source: Pathogens. 2020 Jan 3;9(1):40. doi: 10.3390/pathogens9010040 (PMC7168595; doi:10.3390/pathogens9010040)
Supplement: Supplementary file 1 [file pathogens-09-00040-s001.zip › pathogens-662765-supplementary/Supplement1_rev.pdf]

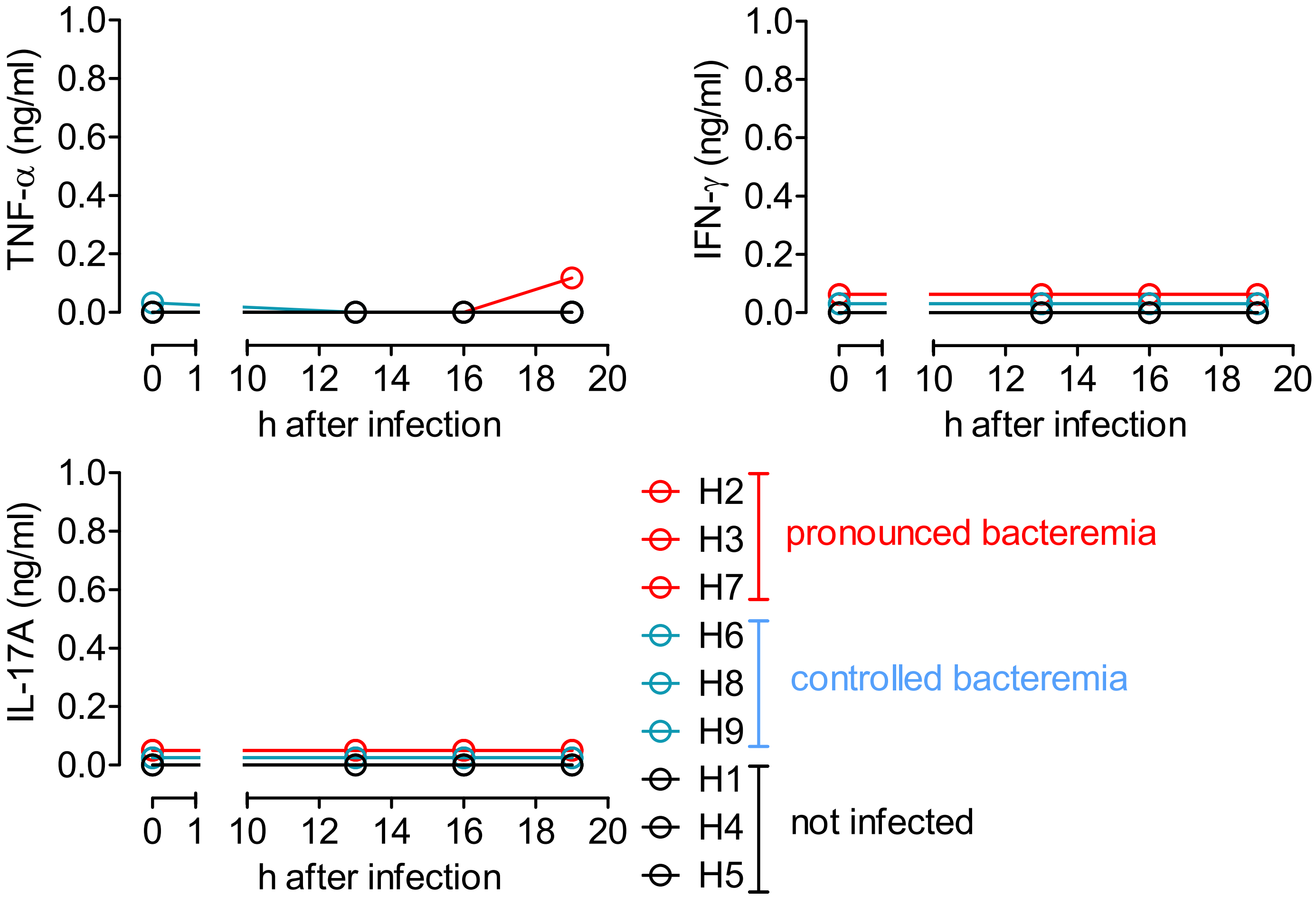

Figure S1. Levels of TNF-α, IFN-γ and IL-17A in sera of experimentally infected piglets. Cytokine levels in serum measured by ELISA after intravenous infection with *S. suis* strain 10 (*cps2*, n=6) and in non-infected control animals (n=3). Note that earlier time points than 13 hpi could not be taken due to the experimental setup.
