# Supplementary material for: Analysis of Porcine Pro- and Anti-Inflammatory Cytokine Induction by S. suis In Vivo and In Vitro
Source: Pathogens. 2020 Jan 3;9(1):40. doi: 10.3390/pathogens9010040 (PMC7168595; doi:10.3390/pathogens9010040)
Supplement: Supplementary file 1 [file pathogens-09-00040-s001.zip › pathogens-662765-supplementary/Supplement2_proof.pdf]

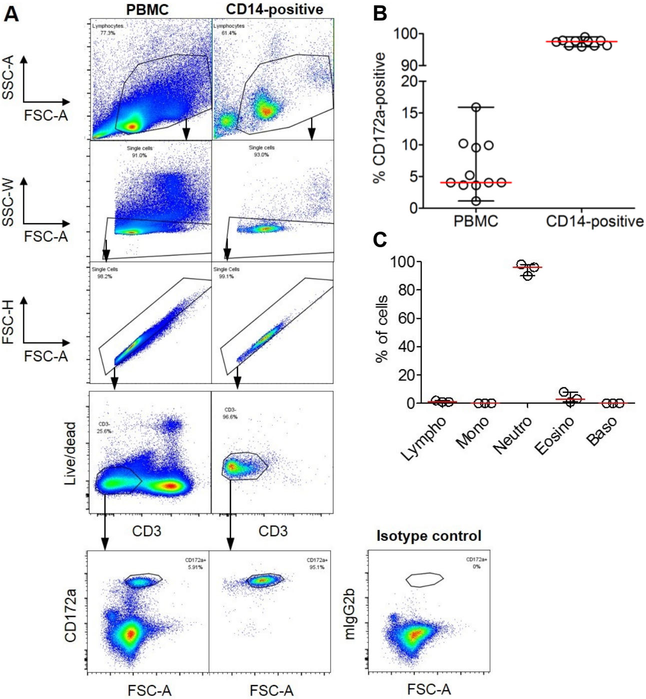

Figure S2. Purity of separated CD14-positive monocytes and granulocytes. (A) Flow cytometric analysis of PBMCs and CD14-positive cells isolated from whole blood stained for CD3 and CD172a. Representative flow cytometry plots show gating strategy (debris exclusion → doublet exclusion → doublet exclusion → live cells/CD3-neg → CD172a-pos) of CD3-negative CD172a-positive cells. (B) Purity of CD14-positive fraction shown as frequency of CD172a-positive cells relative to the CD3-negative parent population. n=10-11, bars show mean with standard deviation. (C) Purity of neutrophil granulocyte fraction shown as frequency of monocytes, lymphocytes and granulocytes in blood pellet after Ficoll density gradient centrifugation, determined by Diff Quik® staining and microscopy.
